# Overlooking Evolution: A Systematic Analysis of Cancer Relapse and Therapeutic Resistance Research

**DOI:** 10.1371/journal.pone.0026100

**Published:** 2011-11-17

**Authors:** C. Athena Aktipis, Virginia S. Y. Kwan, Kathryn A. Johnson, Steven L. Neuberg, Carlo C. Maley

**Affiliations:** 1 Department of Psychology, Arizona State University, Tempe, Arizona, United States of America; 2 Deparment of Surger, Center for Evolution and Cancer, Helen Diller Family Comprehensive Cancer Center, University of California San Francisco, San Francisco, California, United States of America; University of Cape Town, South Africa

## Abstract

Cancer therapy selects for cancer cells resistant to treatment, a process that is fundamentally evolutionary. To what extent, however, is the evolutionary perspective employed in research on therapeutic resistance and relapse? We analyzed 6,228 papers on therapeutic resistance and/or relapse in cancers and found that the use of evolution terms in abstracts has remained at about 1% since the 1980s. However, detailed coding of 22 recent papers revealed a higher proportion of papers using evolutionary methods or evolutionary theory, although this number is still less than 10%. Despite the fact that relapse and therapeutic resistance is essentially an evolutionary process, it appears that this framework has not permeated research. This represents an unrealized opportunity for advances in research on therapeutic resistance.

## Introduction

**Figure 1:**
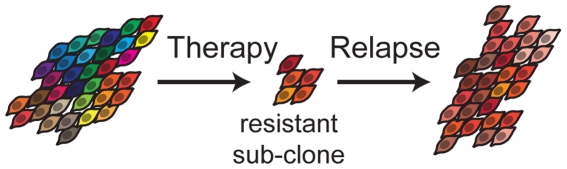
The Evolution of Resistance. An evolutionary view of cancer reveals that therapy selects for resistant cells among an initially heterogeneous population. When the patient relapses, the tumor is composed of a new diverse population of resistant cells generated by further genetic alterations.

Evolutionary theory can provide a functional framework for understanding disease and dysfunction. One example of this is therapeutic resistance in cancer, which is fundamentally an evolutionary process. Neoplasms are genetically [1]–[9] and epigenetically [10] diverse populations of billions to trillions of cells. Therapies apply strong selective pressures to these populations, and when they do not cure the patient, they select for resistant populations of neoplastic cells. When the tumor recurs, it now derives from the resistant cells that survived therapy (see Figure 1), and so application of the same therapy typically has diminished, if any, effect [11], [12]. When tested, the resistant mutations can often be found in the gene targeted by the drug [13]–[23] and are present in tumor samples taken prior to therapy [24], [25]. This shows that therapy did not create the resistance mutations but rather selected the resistant clone from among the standing variation in the cell population at the time of therapy. Every known cancer drug suffers from this problem [26], and it is the primary reason we have not been able to cure cancer. The result is that virtually all cancer deaths are due to therapeutically resistant disease.

Given the magnitude of the problem of therapeutic resistance and fundamentally evolutionary nature of the process, one might expect evolutionary theory and methods to be common in research on therapeutic resistance. However, evolutionary thinking has been strangely absent from research and training in medicine in general [27] and evolutionary terms appear rarely in the medical literature on antibiotic resistance [28] suggesting that evolutionary approaches to therapeutic resistance in cancer might not be very common.

An evolutionary approach to therapeutic resistance in cancer should involve the use of evolutionary theory and the use of methods that take into account the evolutionary nature of therapeutic resistance. This includes (but is not limited to):

### 1. Using evolutionary theory

#### Using evolution to explain how resistance occurs

There are other popular views of resistance that probably play some role in the failure of therapies including: (1) change in phenotype without change in heritable information (such as the [epi]genotype, (2) failure to kill cancer stem cells, (3) too low a dose (toxicity limitations), (4) failure to deliver drug to all the cells (refugia), or (5) between-patient differential sensitivity. However, these other mechanisms of resistance do not lead to the diminishing effectiveness of a drug and so are less clinically problematic than selection for resistant subclones in the tumor. The acquisition of therapeutic resistance is a fundamentally evolutionary process and natural selection is at work during treatment and competitive release (the subsequent increase in population size of the resistance clone because of the removal of competitors), even if other explanations of resistance play some role.

#### Using evolution as a fundamental theoretical framework

The theory of cancer is a theory of evolution among somatic cells of the body [29]. An evolutionary approach to therapeutic resistance depends on the recognition of the population dynamics of somatic cells and selection at that level.

### 2. Measuring evolution

#### Examining within-patient/within-tumor heterogeneity

Because evolution is defined as changes in allele frequencies in a population, measuring within-tumor genetic heterogeneity allows for the study of evolutionary dynamics.

#### Measuring cell fitness

Differential survival and reproduction is necessary for natural selection. Measuring cell survival and proliferation can therefore help researchers understand the evolutionary dynamics underlying therapeutic resistance.

### 3. Detecting resistant cells

#### Looking for resistant cells rather than sensitive cells

If researchers are looking only for therapeutic response or sensitivity, they may find a drug that results in shrinkage of the tumor, but if there are resistant cells, relapse will result. It is therefore necessary to know whether there are cells resistant to the therapy prior to application of that therapy in order to minimize the likelihood of relapse/therapeutic resistance

#### Collecting and analyzing a post-therapy sample

A post-therapy sample is necessary to determine how the cell population responded to the selective pressure of therapy.

In this paper, we assessed the extent to which evolutionary theory and methods have been used in research on therapeutic resistance and relapse in cancer.

## Methods

### Analysis of Abstracts

To explore the extent to which evolutionary approaches have been applied in cancer research, in Study 1 we conducted an automated analysis identifying all papers from the PubMed database (from 8/1/1915-10/11/2010) that contained ‘cancer’ in the title/abstract and ‘relapse’ or ‘resistance’ in the title, and that had available English-language abstracts; this yielded 6,228 abstracts. We then employed a PERL script to count the number of entries with evolution-related terms in the title or abstract, regardless of the case of those words. These titles and abstracts were then individually read by Aktipis and Maley to check that these terms were used to refer to Darwinian evolutionary processes in therapeutic resistance. We excluded titles/abstracts that: 1) referred to the ‘evolution’ of a model, paradigm or treatment practice, 2) simply used the term in the name of the institution, 3) referred to the evolutionary conservation of a physiological mechanism, or 4) referred to the evolutionary history of a species. A linear regression of the frequency of evolutionary terms over time was carried out with the R package, weighting each data point by the inverse of the binomial variance, 1/sqrt(p*(1-p)/n). To avoid zero variance in years with no abstracts using evolutionary terms, a sliding window of 3 years was used to sum the number of abstracts with evolutionary terms as well as the (denominator of) the number of abstracts in those years, to estimate the variance for each year [30].

### Analysis of Papers

In Study 2, we selected the 10 most recent papers (as of 10/1/10) from each of three databases (PubMed, ISI, Medline) that contained the terms “therapeutic resistance” or “relapse” in TITLE and “cancer” in the ABSTRACT. We excluded duplicates, papers not addressing relapse in cancer, and conference abstracts, for a total of 22 unique papers (Table 1) [31]–[52]. Aktipis and Maley then coded these papers for the presence or absence of components of an evolutionary approach to therapeutic resistance. Article coding criteria correspond to the components of an evolutionary approach described in the introduction.

**Table 1:** Papers coded for evolutionary terms and methods in study 2.

Article title	Journal title
Predicting Post-External Beam Radiation Therapy PSA Relapse of Prostate Cancer Using Pretreatment MRI.	International Journal of Radiation Oncology Biology Physics
A hypothesis and theoretical model speculating the possible role of therapy mediated neoplastic cell loss in promoting the process of glioblastoma relapse.	Journal of Theoretical Biology
DNA repair gene expression and risk of locoregional relapse in breast cancer patients.	International Journal of Radiation Oncology Biology Physics
Involved field radiotherapy for locally advanced non-small cell lung cancer: isolated mediastinal nodal relapse.	Lung Cancer
Minimizing early relapse and maximizing treatment outcomes in hormone-sensitive postmenopausal breast cancer: efficacy review of AI trials.	Cancer Metastasis
Thoracoscopic approach in the treatment of breast cancer relapse in the internal mammary lymph node.	Interactive CardioVascular and Thoracic Surgery
Melanoma sentinel node biopsy and prediction models for relapse and overall survival.	British Journal of Cancer
HIF-1alpha is an unfavorable determinant of relapse in gastric cancer patients who underwent curative surgery followed by adjuvant 5-FU chemotherapy.	International Journal of Cancer
Impact of Epidermal Growth Factor Receptor Expression on Disease-Free Survival and Rate of Pelvic Relapse in Patients With Advanced Cancer of the Cervix Treated With Chemoradiotherapy.	American Journal of Clinical Oncology
Does a tertiary Gleason pattern 4 or 5 influence the risk of biochemical relapse after radical prostatectomy for clinically localized prostate cancer?	Scandinavian Journal of Urology and Nephrology
Mantle cell lymphoma in relapse: the role of emerging new drugs.	Current Opinion in Oncology
Epigenetic alterations in disseminated neuroblastoma tumour cells: influence of TMS1 gene hypermethylation in relapse risk in NB patients.	Journal of Cancer Research and Clinical Oncology
Vascular endothelial growth factor (VEGF) and endothelial nitric oxide synthase (NOS3) polymorphisms are associated with high relapse risk in childhood acute lymphoblastic leukemia (ALL).	Clinica Chimica Acta
Intermediate filament dynamics and breast cancer: aberrant promoter methylation of the Synemin gene is associated with early tumor relapse.	Oncogene
Pattern of relapse in surgical treated patients with thoracic esophageal squamous cell carcinoma and its possible impact on target delineation for postoperative radiotherapy.	Radiotherapy & Oncology
High dose chemotherapy as salvage treatment for unresectable late relapse germ cell tumors.	Journal of Urology
Prolonged relapse-free survival in two patients with an isolated brain metastasis from epithelial ovarian carcinoma.	Journal of Clinical Oncology
Lymphopenia assessed during routine follow-up after immunochemotherapy (R-CHOP) is a risk factor for predicting relapse in patients with diffuse large B-cell lymphoma.	Leukemia
IKZF1 deletions predict relapse in uniformly treated pediatric precursor B-ALL.	Leukemia
Prolonged tamoxifen treatment increases relapse-free survival for patients with primary breast cancer expressing high levels of VEGF.	European Journal of Cancer
Donor lymphocyte infusion for leukemia relapse after hematopoietic stem cell transplantation.	ScienceDirect - Transfusion and Apheresis Science
Improved survival of multiple myeloma patients with late relapse after high-dose treatment and stem cell support, a population-based study of 348 patients in Denmark in 1994–2004.	European Journal of Haematology

These 22 recent papers [32]–[53] met the criteria for inclusion in study 2 and were coded for their use of evolutionary methods and theory.

## Results

### Abstract analysis results

**Figure 2:**
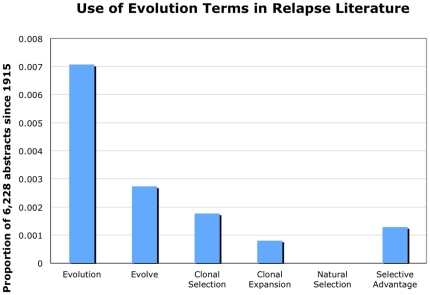
Use of evolution terms in relapse literature. Proportion of abstracts on therapeutic resistance/relapse using each evolution term in 6,228 PubMed abstracts going back to 1915.

**Figure 3:**
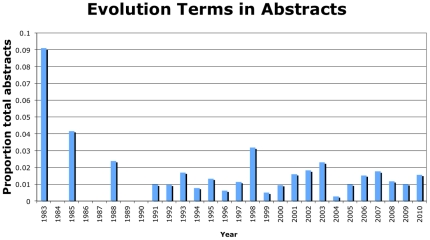
Evolution terms in abstracts. Proportion of abstracts each year on therapeutic resistance/relapse using at least one evolution term out of 6,228 PubMed abstracts going back to 1915 (there was no use of evolution terms before 1983).

Fewer than 1% of papers included any single evolution term. ‘Evolution’ was the most common evolution term (44 papers), with ‘evolve’ (17 papers), ‘clonal selection’ (11 papers), ‘selective advantage’ (8 papers) and ‘clonal expansion’ (5 papers) also appearing (Figure 2). Interestingly, the term ‘natural selection’ was not found in any of the analyzed abstracts. Our analysis of the use of evolution terms over time shows no use of these terms until 1983 (Figure 3). For comparison, the evolutionary theory of cancer was published in *Science* in 1976 [53]. Further, there has been little change in the use of these evolution terms over time, though there is high variability in early years due to few overall papers being published on therapeutic resistance (Figure 3). Regression analysis weighted by the variance for each year shows that the slope is not is not significantly different from 0 (slope = 6.9×10^−5^, std. err.  = 2.6×10^−4^, p = 0.79), indicating that the frequency use of evolution terms in the therapeutic resistance literature has not changed since 1983. Our analyses reveal that evolutionary framing of therapeutic resistance in published articles is still rare. The journals that included the largest numbers of abstracts with evolution terms were *Cancer Research* and *PNAS* (Table 2), but those journals tended to have many articles on therapeutic resistance. The *Journal of Theoretical Biology* stands out as the journal with the highest relative frequency of evolutionarily informed articles on therapeutic resistance (50%, 2 of 4), though the small numbers involved should caution against drawing strong inferences from this data.

**Table 2:** Journals in which evolution terms appeared in at least two abstracts.

Journal	# of evolution term in abstract	Frequency among abstracts on therapeutic resistance in that journal
1. Cancer Research	5	0.0117
2. Proceeding of the National Academy of Sciences of the United States of America	4	0.0645
3. Leukemia	3	0.1500
4. International Journal of Cancer	3	0.0156
5. Clinical Cancer Research	3	0.0155
6. British Journal of Cancer	3	0.0189
7. Journal of Theoretical Biology	2	0.5000
8. International Journal of Oncology	2	0.0323
9. Current Medicinal Chemistry	2	0.1333
10. Carcinogenesis	2	0.0606
11. Breast Cancer Research and Treatment	2	0.0227
12. Biomedical Central Cancer	2	0.0606

The rightmost column was calculated by dividing the number of cancer therapeutic resistance/relapse abstracts with evolution terms in that journal (middle column) by the total number of abstracts on cancer therapeutic resistance/relapse in that journal (out of 6,228 across journals).

### Analysis of full articles

We evaluated each of the 22 unique articles for use of evolutionary theory and methods. We found little evidence that evolution is used a theoretical framework for understanding therapeutic resistance, little evidence for the use of methods for measuring evolution, and mixed evidence for the use of methods to detect resistant cells.

#### 1. Using evolutionary theory

**Figure 4:**
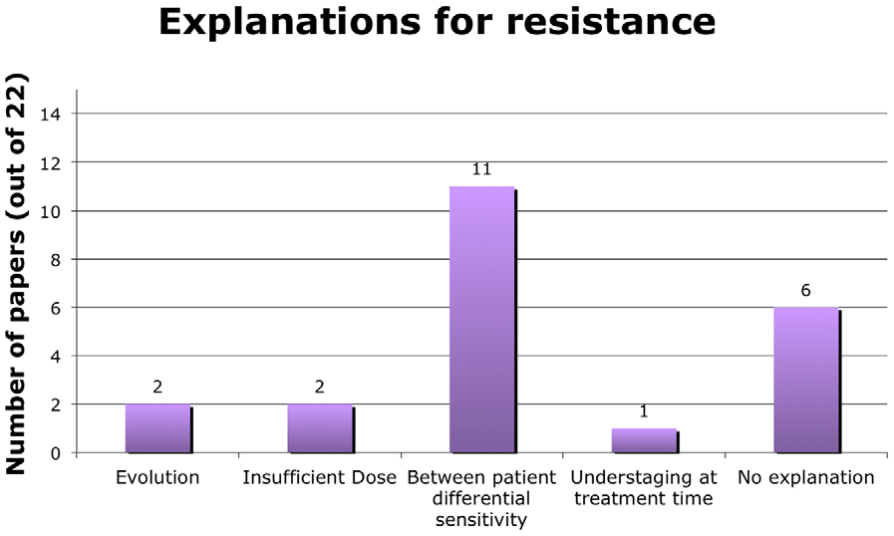
Explanations for resistance. Number of papers using each explanation for resistance out of 22 coded papers.

Using evolution to explain how resistance occurs- Only two papers used evolution as an explanation for relapse/resistance (Figure 4). One of these was a paper about leukemia and the other about neuroblastoma. Other explanations for resistance given in the 22 papers were insufficient dose (2 papers) and understaging at the time of treatment (1 paper). Eleven papers ascribed resistance to between-patient differential sensitivity, which is simply a restatement of the results that some patients appeared to be cured while others relapsed (rather than a true explanation). Six of the 22 papers (27%) did not provide any explanation for resistance.

Using evolution as a fundamental theoretical framework- Two papers used evolution as a theoretical framework for understanding the results. One paper used the cancer stem cell hypothesis. Nineteen papers did not use any theory to interpret their results.

#### 2. Measuring evolution

**Figure 5:**
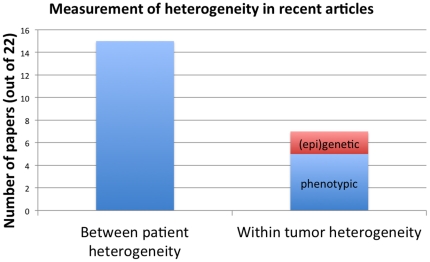
Measurement of heterogeneity in recent articles. Numbers of papers measuring each type of heterogeneity out of 22 coded articles. Only 2 of 22 papers measured epigenetic or genetic within-tumor heterogeneity.

Examining within-patient/within-tumor heterogeneity- Variation is essential for evolution; assessing within-tumor genetic (or epigenetic) heterogeneity thereby allows for the study of evolutionary dynamics. Only two of the articles, however, measured epigenetic or genetic within-tumor heterogeneity (Figure 5). Five papers described phenotypic heterogeneity among cells, which can be done easily with standard immunohistochemical assays. However, phenotypic heterogeneity among cells was not measured with respect to selection on those phenotypes.

Measuring cell fitness- Only one paper of the 22 articles measured cell survival/proliferation differences, in this case between an experimental model of resistant and sensitive cell lines [54].

#### 3. Detecting resistant cells

Looking for resistant cells rather than sensitive cells- The majority of papers (68%, 15 of 22) either discussed or measured resistance/survival of neoplastic cells. Only 18% (4 of 22) focused on response/sensitivity to the therapy.

Collecting and analyzing a post-therapy sample- Despite the fact that a post-therapy sample is necessary to determine how the cell population responded to the selective pressure of therapy, only 2 articles reported collecting a post-therapy sample. These two papers were both in the journal *Leukemia*, and one of these two papers used evolution as an explanation for therapeutic resistance and as an overall framework for the paper. None of the reviewed papers measured efficacy of the initial treatment after relapse.

## Discussion

### Summary of data

Our literature search revealed some interesting observations regarding therapeutic resistance and relapse research. Particularly striking is that studies often overlook therapeutic resistance/relapse as a fundamentally evolutionary process. As of October 11, 2010 a total of 6,228 articles published met the search criteria. Of those articles, only 85 used evolution terms. The proportion of papers on therapeutic resistance/relapse using evolution terms in these abstract has remained essentially unchanged over time since 1983, at approximately 1% (Figure 2). In contrast, Antonovics et. al. [28], found that the overall use of the word “evolution” in journal articles and grant proposals has been increasing since 1991. This suggests that the infrequent use of evolutionary terms in therapeutic resistance research may be due to barriers that are specific to evolutionary thinking in cancer rather than general barriers to using evolution in research.

Nevertheless, we did see slightly higher levels of use of evolutionary approaches in the 22 articles we coded comprehensively as compared to the analyzed abstracts. We found evidence that researchers attempted to measure resistant cells, with the majority of papers focusing on resistant cells rather than sensitive cells, and a small number of papers (two) reporting taking post-therapy samples. However, the focus on resistance rather than drug sensitivity is probably due to the fact that we only selected papers that mentioned resistance or relapse in the title. If we had included all papers on cancer therapy, many more would focus on initial response to the therapy. We found a few instances of researchers using methods for measuring evolution, with only two papers measuring within-tumor heterogeneity (Figure 4) and one measuring cell fitness.

We also found limited evidence of researchers using evolutionary theory. Two of the 22 papers we coded comprehensively (9%) used evolution as a framework and explanation (Figure 5), which suggests that our abstract analysis may be slightly underestimating the number of therapeutic resistance papers using evolution. Indeed, only one of these two papers used an evolution term (“selective advantage”) in the abstract, and neither paper used an evolution term in the title. Therefore, it might be the case that papers using evolution as a framework or explanation do not necessarily note this in the title or abstract and so would have been missed by our analysis of abstracts.

Strikingly, only 4 of 22 (18%) of papers included an explanation (selection for resistance or insufficient dose) for the phenomenon under study (Figure 5). Several papers [11], ascribed resistance to the fact that some patients were cured and others were not, but this does not constitute an explanation for why this occurred. Ascribing relapse to under-staging (as 1 paper did) also does not explain why late stage patients were likely to relapse. This lack of explanation is worrisome in that it is difficult to make scientific progress if no one asks why therapeutic resistance occurs. Without an explanation for the results, there is no theoretical framework for generating follow-up hypotheses and study designs.

Finally, 19 of the 22 papers employed no apparent overall theoretical framework, let alone any specific explanation for resistance. This finding suggests that it is not the case that evolutionary theory is unsuccessfully competing with other theories of therapeutic resistance, but rather that there is a dismaying absence of theory in the literature on therapeutic resistance and relapse in cancer.

In all, these findings reveal the under-utilization of the evolutionary perspective for the feature of cancer for which the evolutionary approach is arguably most relevant—acquired therapeutic resistance.

### Why isn't evolution used as a framework?

Despite the fact that relapse and therapeutic resistance is essentially an evolutionary process, our analysis shows that this framework has not permeated research. This is likely due to a variety of factors including the use of methods that do not allow for collecting evolutionary data, a lack of evolutionary training in medical education, and psychological barriers to evolutionary thinking.

#### Methodological barriers

Science is limited by what we can observe with the current tools. We can only see what is under the proverbial lamppost. Cytological staining of chromosomes in mitotic spreads allowed early researchers to observe sequential accumulation of genomic lesions in leukemias back in the 1960's, which led directly to Peter Nowell's formulation of the evolutionary theory of carcinogenesis [55]. However, much of the last few decades of cancer research has been dominated by the assays of molecular biology that homogenize a tissue sample in order to measure the average protein/RNA/DNA values in the population of cells. These methods obscure the heterogeneity among cells in a neoplasm and make it difficult to study the evolutionary dynamics within those neoplasms. Furthermore, most cancer research has been based on cross-sectional study designs, making it difficult to study changes in a neoplasm over time. This is because most neoplasms are removed when detected, and so cannot be followed over time. Similarly, most animal studies utilize a serial sacrifice design, making it impossible to observe evolution over time within the same neoplasm. Clinically, the acquisition of post-therapy biopsies has been limited because doctors have been reluctant to subject patients to an invasive procedure to collect a biopsy when a tumor recurs. It is important to note however, that both medical oncologists and the internal review boards (IRBs) that approve of research studies, over-estimate patients' anxiety associated with undergoing a research-related biopsy, and under-estimate patients' willingness to accept risks associated with those biopsies [56]. This suggests that patients are more willing to provide longitudinal biopsies than has been assumed, which would facilitate the study and management of therapeutic resistance.

In contrast, progress in the treatment of chronic myeloid leukemia (CML) is notable and has been due to the relative ease of gathering longitudinal samples of blood, thereby enabling researchers to study the dynamic, evolutionary nature of CML. Because cytology reveals tumor heterogeneity at the single cell level, researchers were able to recognize the driving mutation in CML (the BCR-ABL gene fusion) [55], [57], develop a successful drug (*imatanib*) to target that lesion [58], observe the selective effects of imatanib treatment [59], [60] and, with that knowledge in hand, develop second-line drugs (e.g., dasatinib) that work on imatanib- resistant CML [22], [61]. Rapid progress in CML illustrates how studying the evolutionary process accelerates research and leads to treatments for even therapeutically resistant cancers. It is perhaps not surprising that the 2 (of 22) papers that collected post-therapy samples were published in the journal *Leukemia*.

Fortunately, improved study designs and technologies are making it easier to study the evolutionary dynamics of other cancers as well. Taking multiple biopsies, or assaying single cells, from a solid tumor enables one to detect cellular diversity within the neoplasm [7], [9], [62], and to generate phylogenetic inferences of the genetic events in the history of that neoplasm [63]–[65]. Deep sequencing is becoming common and is revealing the presence of genetic diversity within neoplasms [66], [67]. This trend should continue as more single cell assays are developed. Finally, animal studies may be improved by taking longitudinal biopsies, rather than sacrificing animals at different time points, though the wounding from the biopsy removal may perturb the system.

#### Educational opportunities

There is a great opportunity to amplify the effectiveness of research and treatment with better penetration of evolutionary approaches to cancer. Although a theoretical understanding of cancer as an evolutionary process has been generally accepted in cancer biology, our literature review shows that research on therapeutic resistance grounded in evolutionary theory has been largely neglected to date. Furthermore, evolutionary thinking has not yet been incorporated into medical education, although this can be overcome by developing a clear set of training goals [27] and incorporating them into medical school curricula.

Because evolutionary medicine is not currently a core component of medical education, a great deal of attention has recently been paid to the question of how to more effectively increase exposure to evolutionary approaches in medical training. This includes a recent Sackler Colloquium on the topic and a paper, co-authored by a large number of evolutionary medicine experts, entitled, “Making evolutionary biology a basic science for medicine” [27]. In this paper, the authors provide a set of general recommendations and specific learning objectives for effectively incorporating evolutionary theory into medical education. These include pre-med competencies such as understanding natural selection, the role of mutation and drift, the use of the comparative method and the role of tradeoffs. They also lay out a number of medical competencies, which include understanding the use of phylogenetic methods, co-evolution, somatic evolution and the evolutionary origins of senescence.

Despite the current lack of evolutionary training in medical schools, efforts to incorporate evolution biology into medical curricula are being developed at Harvard, Yale and John Hopkins [27]. Also, the National Evolutionary Synthesis Center (NESCent) is supporting a working group on the topic “Infusing Medical Education with Evolutionary Thinking,” with a number of goals including evaluating present evolutionary education in medical schools, developing evolutionary medicine curricula and evaluating the effectiveness of novel educational interventions on student learning and clinical problem solving. Given the fundamental role of evolutionary theory in cancer biology, and the lack of its use in contemporary research, we strongly support these efforts.

#### Psychological barriers

Understanding the tendencies and biases in human cognition may help us to identify psychological barriers to evolutionary thinking in cancer [68]. Some of these psychological barriers may apply to evolutionary thinking in general. Thinking in evolutionary terms is not intuitive, even for the well informed [69], [70]. Also, many lay people and healthcare professional may react negatively to interventions framed in terms of evolution. General barriers such as these may be addressed by using familiar analogies to explain evolutionary processes, such as the evolution of antibiotic resistance or pesticide resistance.

Other barriers may be more specific to evolutionary thinking in the domain of cancer. To address these specific psychological barriers, our research team is currently investigating misconceptions about cancer (held by medical students and medical professionals) that reflect a lack of evolutionary thinking. One of these misconceptions is the tendency to essentialize tumors, whereby one views a cancerous tumor as an entity with some internal property or essence that gives rise to its outward appearance [68], [71], [70]. However, cancerous tumors are neither unitary nor static, but collections of mutable cells with differential capacities for proliferation. There are over 200 different kinds of cancer currently recognized and it is important to understand that clinically advanced tumors are nearly always heterogeneous populations of differentially mutated cells. Just as essentialist thinkers have difficulty conceptualizing a species as being a collection of unique individuals rather than a homogenous group [69], it may also be counterintuitive for some to think of tumors as being a collection of heterogeneous and mutable cells. An essentialist bias may make it difficult for researchers to study how neoplastic cell populations change in response to the selective pressures of therapy, and thus interfere with the development of strategies to prevent or manage therapeutic resistance.

### Conclusions

Future research should address the barriers that impede the progress of applying the evolutionary approach to cancer research and treatment. The problems may lie in the unfamiliarity of evolutionary principles (e.g., created by inadequate training and education), the dominance of non-evolutionary approaches used by grant and manuscript reviewers, or in psychological barriers to thinking about cancer in evolutionary terms. Our analyses show that most cancer research on therapeutic resistance has not utilized an evolutionary approach. Of course, not all research on acquired therapeutic resistance has to focus on the change in the cell population in response to therapy. For example, the molecular mechanism of resistance could be studied without reference to the evolutionary dynamics that produced it. However, the components of an evolutionary approach that we identified are almost entirely absent from the literature on therapeutic resistance and relapse. This is surprising given that acquired therapeutic resistance is one of the clearest cases of the relevance of evolutionary theory in cancer. Grounding cancer research and treatment in the principles of evolutionary theory may elicit new and more successful interventions, as illustrated by progress in the treatment of CML.
